# NAPSB as a predictive marker for prognosis and therapy associated with an immuno-hot tumor microenvironment in hepatocellular carcinoma

**DOI:** 10.1186/s12876-022-02475-8

**Published:** 2022-08-20

**Authors:** Yu-Mei Ning, Kun Lin, Xiao-Ping Liu, Yang Ding, Xiang Jiang, Zhang Zhang, Yu-Ting Xuan, Li Dong, Lan Liu, Fan Wang, Qiu Zhao, Hai-Zhou Wang, Jun Fang

**Affiliations:** 1 Department of Gastroenterology, Zhongnan Hospital of Wuhan University, No.169 Donghu Road, Wuhan, 430071 Hubei Province China; 2 Hubei Clinical Center and Key Lab of Intestinal and Colorectal Diseases, Wuhan, China; 3 Department of Pathology, Zhongnan Hospital of Wuhan University, Wuhan, China; 4 Renmin Hospital of Huangmei County, Huanggang, China

**Keywords:** NAPSB, Hepatocellular carcinoma, Tumor microenvironment, Immunotherapy, Chemotherapy

## Abstract

**Background:**

Napsin B Aspartic Peptidase, Pseudogene (NAPSB) was associated with CD4 + T cell infiltration in pancreatic ductal adenocarcinoma. However, the biological role of NAPSB in hepatocellular carcinoma (HCC) remains to be determined.

**Methods:**

The expression of NAPSB in HCC as well as its clinicopathological association were analyzed using data from several public datasets. qRT-PCR was used to verify the relative expression of NAPSB in patients with HCC using the Zhongnan cohort. Kaplan–Meier analyses, and univariate and multivariate Cox regression were conducted to determine the prognosis value of NAPSB on patients with HCC. Then enrichment analyses were performed to identify the possible biological functions of NAPSB. Subsequently, the immunological characteristics of NAPSB in the HCC tumor microenvironment (TME) were demonstrated comprehensively. The role of NAPSB in predicting hot tumors and its impact on immunotherapy and chemotherapy responses was also analyzed by bioinformatics methods.

**Results:**

NAPSB was downregulated in patients with HCC and high NAPSB expression showed an improved survival outcome. Enrichment analyses showed that NAPSB was related to immune activation. NAPSB was positively correlated with immunomodulators, tumor-infiltrating immune cells, T cell inflamed score and cancer-immunity cycle, and highly expressed in immuno-hot tumors. High expression of NAPSB was sensitive to immunotherapy and chemotherapy, possibly due to its association with pyroptosis, apoptosis and necrosis.

**Conclusions:**

NAPSB was correlated with an immuno-hot and inflamed TME, and tumor cell death. It can be utilized as a promising predictive marker for prognosis and therapy in HCC.

**Supplementary Information:**

The online version contains supplementary material available at 10.1186/s12876-022-02475-8.

## Introduction

Hepatocellular carcinoma (HCC) is the sixth most frequent malignancy worldwide and the third leading cause of cancer-related deaths, accounting for almost 90% of primary liver cancer [1]. Systemic treatments are the important options for patients with HCC [2], and emerging immunotherapies involving the use of immune checkpoint inhibitors (ICIs) are currently the focus of research in many advanced cancers [3–5]. However, both systemic chemotherapy and immune checkpoint therapy have limitations of response in only some patients [6, 7]. Therefore, it is urgent and beneficial to identify new biomarkers for individualized therapy.

In HCC, the tumor microenvironment (TME) composed of cancer cells, immune cells and extracellular matrix has an immunosuppressive effect, promoting immune tolerance and avoidance [8]. However, recent studies have shown that abundant infiltration of CD8 + , CD4 + , regulatory T cells and dendritic cells (DCs) can shape an inflamed TME to anti-cancer and influence the efficacy of ICIs [9, 10]. Based on the characteristics of the TME, tumors can be divided into hot and cold tumors. Hot tumors are characterized by T cell infiltration, molecular characteristics of immune activation, and response to cancer immunotherapy, while cold tumors are characterized by the opposite [11].

Over the past decade, non-coding RNAs including pseudogene, long non-coding RNA and microRNA have been demonstrated to play crucial roles in TME [12, 13]. Napsin B Aspartic Peptidase, Pseudogene (NAPSB) is a pseudogene that has been identified to be associated with the infiltration of CD4 + T immune cells in pancreatic ductal adenocarcinoma (PDAC) [14]. In HCC, NAPSB was found to be downregulated [15], but its biological role has not been elucidated. Thus, in this study, the potential biological functions of NAPSB were comprehensively explored in HCC, including its differential expression, prognosis value and immunological role. We also reported that high NAPSB expression was related to an immuno-hot TME and sensitive to immunotherapy/chemotherapy possibly on account of affecting pyroptosis, apoptosis and necrosis (PANoptosis) in HCC.

## Materials and methods

### Public data collection

The TIMER database (https://cistrome.shinyapps.io/timer/) was used to analyze expression levels of NAPSB in various cancers. Patients with HCC (n = 369) with the transcriptomic RNA-sequencing data (log2 (fragments per kilobase of transcript permillion mapped reads + 1) value) of The Cancer Genome Atlas (TCGA)-LIHC cohort were obtained from University of California Santa Cruz Xena (https://xenabrowser.net/datapages/). Meanwhile, the LIRI-JP (n = 231) cohort retrieved from the International Cancer Genome Consortium (ICGC) database (https://icgc.org/) was chosen for primary external validation. In addition, we used multiple cohorts from Gene Expression Omnibus (GEO) (https://www.ncbi.nlm.nih.gov/geo/), including GSE55092, GSE54236 and GSE121248, to verify the relative expression of NAPSB in HCC and normal tissues.

Two immunotherapy-related cohorts, GSE78220 and GSE91061 (melanoma), were downloaded from the GEO database. GSE104580, a dataset of transcatheter arterial chemoembolization (TACE) for patients with HCC was also downloaded.

### Tissue specimens acquisition

Thirteen HCC tissues and paired adjacent normal tissues were obtained from the Zhongnan Hospital of Wuhan University between February 2021 and September 2021 following patient informed consent. The protocols used in the study were approved by the Medical Ethics Committee of the Zhongnan Hospital of Wuhan University (grant no. 20200110).

### Quantitative reverse transcription polymerase chain reaction (qRT-PCR) assays

Total RNA was extracted from HCC and paired adjacent normal tissues using TRIZOL reagent (Invitrogen, Carlsbad, CA, USA). RNA quantity was determined by NanoDrop2000c (Thermo Scientific, Waltham, MA, USA). For qRT-PCR, 1 μg RNA was reverse transcribed to cDNA using a Reverse Transcription Kit (Toyobo, Osaka, Japan). The qRT-PCR assays were conducted on LightCycler® 96. Target gene expression was normalized against GAPDH. The primer sequences were:

NAPSB-Forward: CATCCAGTTTGCTCAGGGT;

NAPSB-Reverse: TCGAAGACGGTCACATACGC;

GAPDH-Forward: CCCCAGCAAGAGCACAAGAG;

GAPDH-Reverse: GCACAGGGTACTTTATTGATGGTAC.

### Immunohistochemistry (IHC)

Liver tissues were fixed in 10% neutral-buffered formalin (Sigma-Aldrich, USA) and embedded in paraffin. Tissue sections were sliced from paraffin blocks into 4-μm-thick slices. The slices were further used for IHC test. The IHC staining was conducted in the NAPSB-high and NAPSB-low tissues based on the qRT-PCR results. The primary antibodies against CD8 (Wuhan JiaYuan Biomedical Engineering Co., Ltd., Wuhan, China) and PD-L1 (Amoy Diagnostics Co., Ltd., Xiamen, China) were utilized. They were performed heat mediated antigen retrieval with EDTA buffer pH 8.0. Images were processed with Image J software, and relative expression was calculated.

### Evaluate the prognostic value of NAPSB

Kaplan–Meier (K-M) analyses, and univariate and multivariate Cox regression were conducted to explore the influence of NAPSB on the survival of patients with HCC using the R package *“survminer”* and *“survival”.* The log-rank test was applied to estimate statistical significance. Overall survival (OS), disease-free interval (DFI) and progression-free interval (PFI) were evaluated (*p*-value < 0.05 as significant).

### Analysis of NAPSB co-expressed genes and differential expressed genes

Genes potentially positively co-expressed with NAPSB were predicted using R software. Those genes with the thresholds *p*-value < 0.01 and |Spearman`s correlation|≥ 0.45 were selected for further analysis. Patients were classified into two groups based on the median NAPSB expression. We screened differentially expressed genes (DEGs) between the NAPSB-high group and the NAPSB-low group using the *“edgeR”* package in the R software. An adjusted p-value < 0.05 and |log2 fold change (FC)|≥ 1.3 was considered significant. We took the intersection of the co-expressed genes and the upregulated DEGs as the genes most related to NAPSB for further analysis.

### Biological function, pathway annotation, gene set enrichment analysis (GSEA) and gene set variation analysis (GSVA)

We conducted gene ontology (GO) and Kyoto Encyclopedia of Genes and Genomes (KEGG, www.kegg.jp/kegg/kegg1.html) [16] pathway analyses to explore the possible biological function of the genes most related to NAPSB (mentioned above) via the R package *“clusterprofiler”*. To investigate the difference in biological process terms in NAPSB subgroups, GSEA was applied using the R package *“clusterProfiler”* and GSVA was applied using the R package *“GSVA”*. The gene sets of “h.all.v7.4.symbols” and “c5.cp.kegg.v7.4.symbols” were downloaded from Molecular Signatures Database (http://www.gsea-msigdb.org/gsea/index.jsp) for GSEA and GSVA, respectively.

### Evaluation of relationship between NAPSB expression and the immunological characteristics of the TME

A total fifty immunomodulators (including major histocompatibility complex (MHC), immunomodulators, chemokines and receptors) were collected from the study of Charoentong et al. [17] (Additional file 1: Supplementary Table 1). We applied the ESTIMATE algorithm to assess the immune scores, stromal scores, estimate scores and tumor purity for each HCC sample [18]. Several algorithms were used to calculate the infiltration levels of tumor-infiltrating immune cells (TIICs) to avoid calculation errors: single sample gene set enrichment analysis (ssGSEA) [19], TIMER [20], CIBERSORT [21], quanTIseq [22], EPIC [23], xCell [24] and MCP-counter [25]. We identified the effector genes of TIICs from previous studies [26, 27] (Additional file 1: Supplementary Table 2). Also, we calculated the steps of cancer-immunity cycle as described previously [28]. Finally, the T cell–inflamed score was calculated as an average value of log2-scale normalized expression of the 18 signature genes [29].

### Unsupervised clustering

Unsupervised clustering was implemented to classify HCC tissues into hot or cold tumors on the basis of hot tumor signature genes according to previous literature [30]. We used the *“ConsensusClusterPlus”* package to perform this algorithm and 1000 repetitions were conducted for guaranteeing the stability of classification [31].

### Calculation of the enrichment scores of various gene signatures and prediction of immunotherapy response

We analyzed the oncogenic pathways that were associated with targeted therapy, and immunotherapy responses according to previous research [26] (Additional file 1: Supplementary Table 3). The enrichment scores of these signatures were calculated using the R package *“GSVA”* [19]. To analyze the efficacy of immunotherapy, two immunotherapy-related cohorts, GSE78220 and GSE91061 (melanoma) were obtained.

### Prediction of chemotherapeutic response

We downloaded the transcriptional expression data and drug response of more than 1000 cancer cell lines from Genomics of Drug Sensitivity in Cancer (GDSC, http://www.cancerrxgene.org/downloads) [32] and Cancer Therapeutics Response Portal (CTRP) [33], respectively. The Spearman correlations between the NAPSB of each cell line and half maximal inhibitory concentration (IC50) of each cell line to particular drugs were calculated. Correlations with adjusted *p*-value < 0.01 were considered significant. In addition, GSE104580 was used to analyze the correlation between NAPSB expression and TACE response in patients with HCC.

### Calculation of the enrichment scores of cell death gene sets

We collected signatures of several forms of cell death, including pyroptosis, apoptosis, necroptosis, autophagy and ferroptosis, from previous literature [34–38] (Additional file 1: Supplementary Table 4). The enrichment scores of these signatures were also calculated using the R package *“GSVA”* as mentioned above.

### Statistical analysis

Statistical analyses were performed using R software (version 4.1.1). Paired Student’s t-test was performed to detect the differential expression of NAPSB in paired HCC and adjacent normal tissues. One-way ANOVA test were used for comparison of multiple groups. Correlations between variables were explored using Pearson or Spearman coefficients. For all analyses, a two-paired *p*-value < 0.05 was considered statistically significant if not noted. Statistical significance was defined as: ns, no significance; *, *p*-value < 0.05; **, *p*-value < 0.01; ***, *p*-value < 0.001; ****, *p*-value < 0.0001.

## Results

### Expression levels analysis and high NAPSB inferred a better prognosis for HCC

**Fig. 1:**
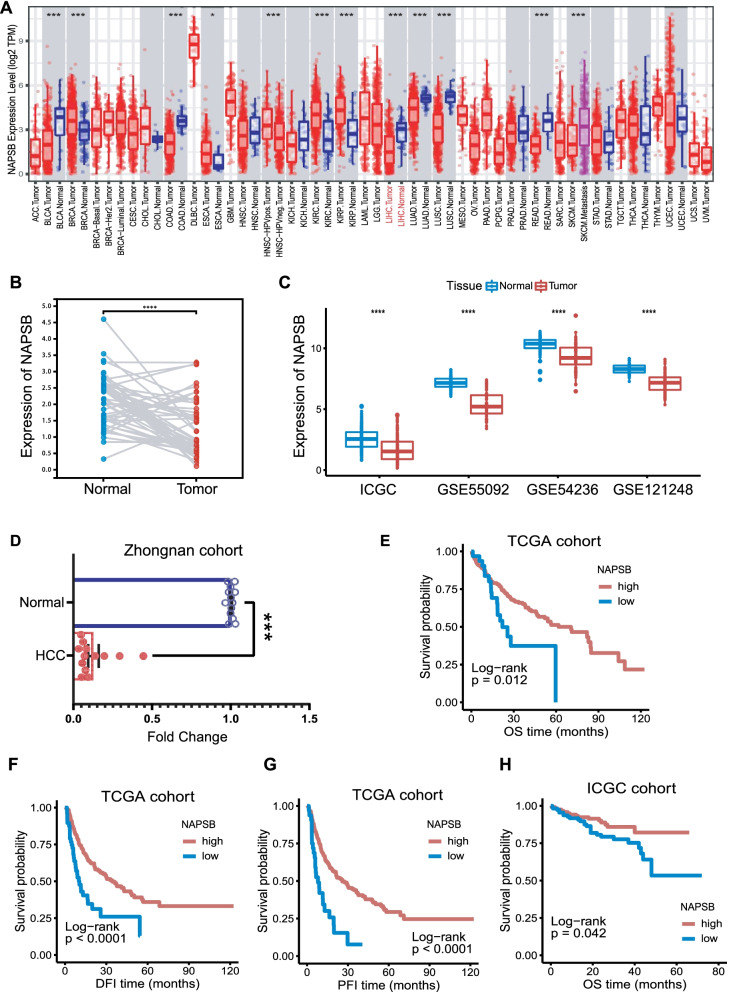
Differential expression and prognosis value of NAPSB in various cancers and liver hepatocellular carcinoma (LIHC). **A** NAPSB expression levels in different tumor types were measured using the TIMER website. **B** Paired Student’s t-test analysis of NAPSB expression in paired samples of TCGA-LIHC. **C** NAPSB expression was significantly higher in normal tissues than in HCC in the ICGC, GSE55092, GSE54236 and GSE121248 cohorts. **D** In the Zhongnan cohort, lower NAPSB expression was observed in HCC compared with adjacent normal tissues (N = 13). The expression of NAPSB was compared with a standard reference control and relative quantities (RQ) were calculated based on the ΔΔCt method. **E–H** Kaplan–Meier analysis of NAPSB expression based on overall survival (OS), disease-free interval (DFI), progression-free interval (PFI) in the TCGA cohort and OS in the ICGC cohort. ns, no significance; *, *p*-value < 0.05; **, *p*-value < 0.01; ***, *p*-value < 0.001; ****, *p*-value < 0.0001

NAPSB transcription levels in different human tumors were shown in Fig. 1A. Compared with adjacent normal tissues, expression of NAPSB in BLCA (bladder urothelial carcinoma), COAD (colon adenocarcinoma), LIHC (liver hepatocellular carcinoma), LUAD (lung adenocarcinoma), LUSC (lung squamous cell carcinoma) and READ (rectal adenocarcinoma) was significantly decreased. For the TCGA-LIHC cohort, we analyzed paired samples by paired Student’s t-test to verify the above results in HCC (Fig. 1B). To fully demonstrate this expression difference, we validated it with multiple datasets, including ICGC, GSE55092, GSE54236 and GSE121248, finding that NAPSB was indeed significantly decreased in HCC tissues (Fig. 1C). Moreover, NAPSB expression was examined in thirteen paired HCC and adjacent normal tissues of the Zhongnan cohort by qRT-PCR, and consistent results were obtained. (Fig. 1D).

The correlation between NAPSB and clinical features of the TCGA and ICGC cohorts were presented in Additional file 2: Supplementary Tables 5 and 6. In addition, K-M survival analysis showed that high expression of NAPSB was linked to better OS than low expression (Fig. 1E), and more significantly associated with longer DFI (Fig. 1F) and PFI (Fig. 1G). The prognostic value for OS was also verified in the ICGC cohort (Fig. 1H). Univariate Cox regression analysis showed that NAPSB expression was significantly associated with better DFI and PFI outcomes (Additional file 3: Supplementary Fig. 1A) and multivariate Cox regression analysis further validated it (Additional file 3: Supplementary Fig. 1B). Therefore, NAPSB expression was beneficial to OS, and could serve as an independent predictor of DFI and PFI of patients with HCC.

### Enrichment analyses inferred NAPSB was related to immune activation

**Fig. 2:**
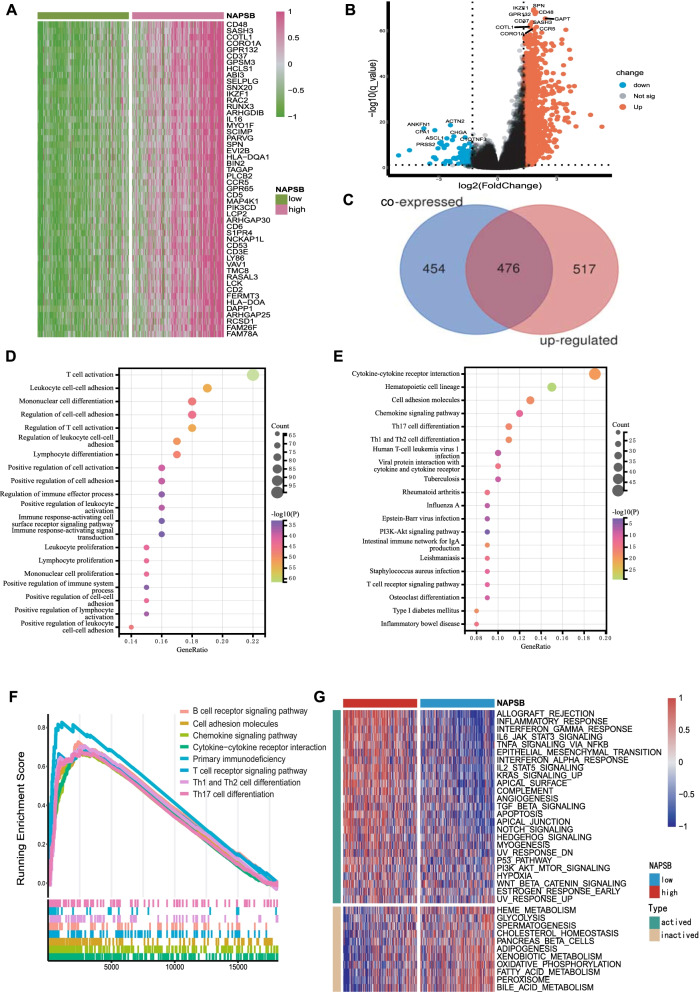
Enrichment analysis of NAPSB biological function in the TCGA-LIHC cohort. **A** The heat map shows the top 50 genes positively related to NAPSB in HCC. **B** Volcano plot of DEGs between the NAPSB-high group and the NAPSB-low group. **C** Venn diagram of co-expressed genes and upregulated DEGs. **D** The top 20 GO terms for the most closely related genes to NAPSB. **E** The top 20 KEGG terms (sourced from the KEGG pathway database: www.kegg.jp/kegg/kegg1.html [16]) for the most closely related genes to NAPSB. **F** GSEA plot shows significant signaling pathways in the patients with HCC (The gene sets of “c5.cp.kegg.v7.4.symbols”). **G** GSVA analysis between NAPSB-high and NAPSB-low expression samples (The gene sets of “h.all.v7.4.symbols”)

Correlation between NAPSB and other genes was analyzed using TCGA-LIHC data, and there were 930 genes significantly associated with NAPSB (*p*-value < 0.01, |Spearman`s correlation|≥ 0.45; Additional file 1: Supplementary Table 7). The correlation of NAPSB with the top 50 co-expressed genes was shown in Fig. 2A, which contained some immune-related molecules like CD48, CD37, IL6 and HLA-DQA1. Meanwhile, DEGs analysis between the NAPSB-high group and the NAPSB-low group showed that there were 993 upregulated genes (adjusted *p*-value < 0.05 and |log2 FC|≥ 1.3; Additional file 1: Supplementary Table 8). The top 10 upregulated genes also contained immune-related molecules, such as CD48, CD37 and CCR5 (Fig. 2B), suggesting that NAPSB may be involved in immunity.

Thereafter, the intersection of co-expressed genes and upregulated DEGs included 476 common genes, which were selected as the genes most closely related to NAPSB (Fig. 2C; Additional file 1: Supplementary Table 9). The GO analysis for these common genes demonstrated they were enriched in processes such as T cell activation, regulation of T cell activation and regulation of immune effector process (Fig. 2D; Additional file 1: Supplementary Table 10). The KEGG analysis showed they were associated with chemokine signaling pathway, Th17 cell differentiation and T cell receptor signaling pathway (Fig. 2E; Additional file 1: Supplementary Table 11). Most biological functions and signaling pathways were immune-related, strongly implying that NAPSB may mediate the TME in HCC.

Even further, we conducted GSEA and GSVA between NAPSB subgroups and also identified many significant pathways related to immunity (Fig. 2F, G; Additional file 1: Supplementary Tables 12 and 13). These findings paralleled the above results.

### NAPSB shaped an immuno-hot and inflamed TME in HCC

**Fig. 3:**
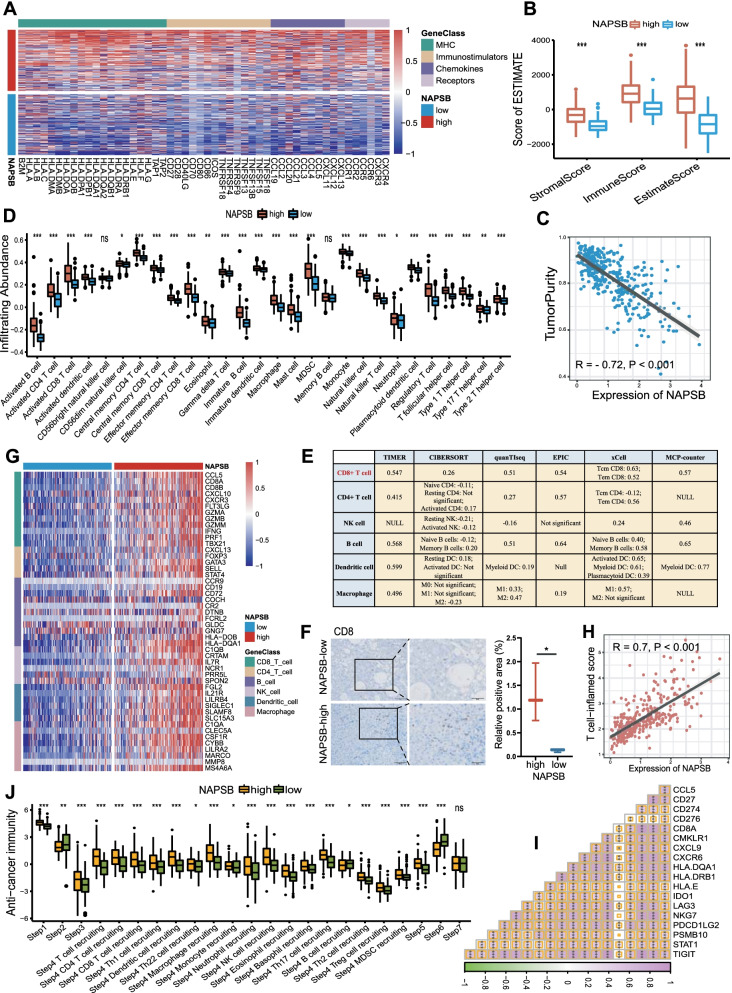
High NAPSB was associated with an inflamed TME among patients with HCC from the TCGA cohort. **A** Expression levels of fifty immunomodulators (MHC, immunostimulators, chemokines and receptors) patients with HCC of the NAPSB-high and NAPSB-low groups. **B** Distribution of stromal score, immune score and estimate score calculated using the ESTIMATE algorithm in the NAPSB-high and NAPSB-low groups. **C** Correlation between NAPSB expression and tumor purity using the ESTIMATE algorithm. **D** Different expression of 28 tumor-associated immune cells calculated with the ssGSEA algorithm between NAPSB subgroups. **E** Correlation between NAPSB expression and the infiltration levels of six types of TIICs (CD8 + T cells, CD4 + T cells, NK cells, B cells, dendritic cells and macrophages), which were calculated using six independent algorithms. **F** Representations (left) and quantification of IHC (right) positive areas of CD8 in NAPSB-high and NAPSB-low HCC tissues. **G** Correlation between NAPSB expression and the effector genes of the above immune cells. **H**, **I** Correlations between NAPSB and the T cell inflamed score, and the individual genes included in the T cell inflamed signature. **J** The activities of the various steps of the cancer-immunity cycle in the NAPSB-high and NAPSB-low groups. ns, no significance; *, *p*-value < 0.05; **, *p*-value < 0.01; ***, *p*-value < 0.001; ****, *p*-value < 0.0001

The immunological role of NAPSB was comprehensively explored subsequently using the TCGA and ICGC cohorts. NAPSB was found to upregulate the expression of critical immunomodulators (including MHC, immunostimulators, chemokines and receptors) (Fig. 3A), which may upregulate the activities of the cancer-immunity cycle subsequently. The ESTIMATE algorithm was applied to calculate the immune score, stromal score, estimate score and tumor purity. We found these scores were significantly increased in the NAPSB-high group (Fig. 3B), while tumor purity was negatively correlated with the expression of NAPSB (Fig. 3C). As for TME immune cell infiltration, almost all immune cells were significantly enriched in the NAPSB-high group (Fig. 3D). Consistently, the infiltration levels of CD8 + T cells, CD4 + T cells, nature killing (NK) cells, B cells, DCs and macrophages were almost positively correlated with NAPSB in six different algorithms, and the CD8 + T cells were the most prominent (Fig. 3E). Based on the IHC staining, a significant increase in CD8 (CD8 + T cell marker) was observed in NAPSB-high HCC tissues of Zhongnan cohort (Fig. 3F). In line with these results, NAPSB was positively correlated with the marker genes of these six major types of immune cells (Fig. 3G). These results suggested NAPSB was associated with an inflamed TME. Even further, we observed the NAPSB expression positively correlated with the T cell inflamed score (TIS) and all genes within this signature (Fig. 3H, I), further confirming its role in shaping a hot inflamed TME. These findings were all verified in the ICGC cohort, and consistent results were obtained (Additional file 4: Supplementary Fig. 2).

Finally, we evaluated the correlation between NAPSB and seven steps of cancer-immunity cycle, which conceptualized the anti-cancer immune response [39]. Overall, in the NAPSB-high group, the activities associated with the majority of the steps in the cycle were notably upregulated (Fig. 3J), including the release of cancer cell antigens (Step 1), cancer antigen presentation (step 2), priming and activation (Step 3), trafficking of immune cells to tumors (Step 4) and infiltration of immune cells into tumors (Step 5). In summary, these data consistently indicated that high expression of NAPSB was to transform a non-inflamed TME into an immuno-hot and inflamed TME, consequently triggering anti-cancer immune response.

### NAPSB highly expressed in hot tumors and may enhance immunotherapy response

**Fig. 4:**
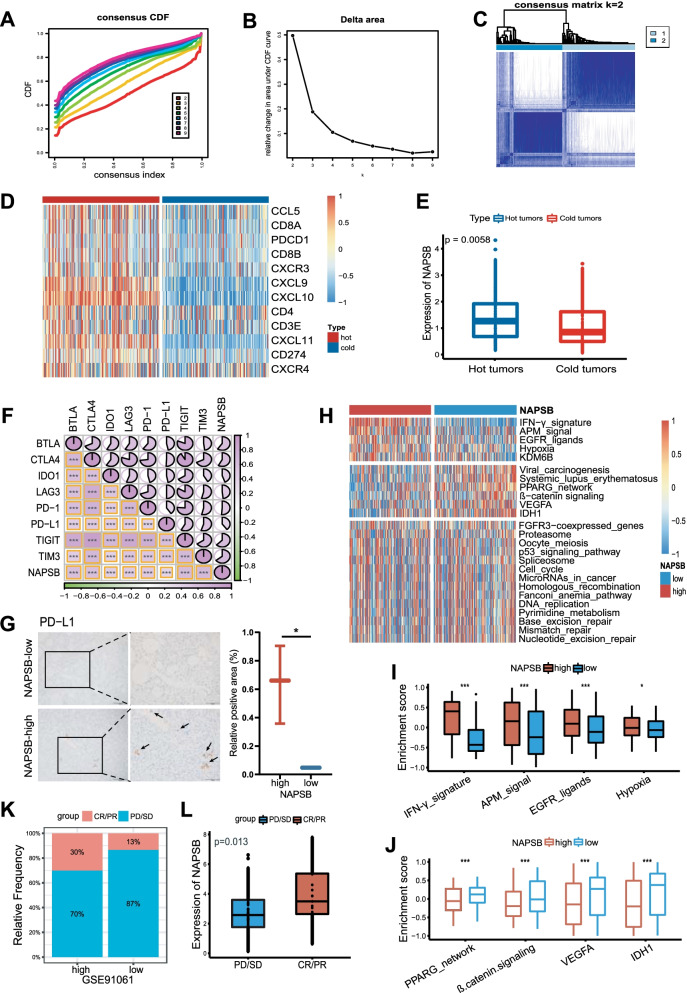
High NAPSB was correlated with a hot tumor status, and improved the response to immunotherapy. **A** Consensus clustering cumulative distribution function (CDF) for k = 2–9. **B** Relative change in area under CDF curve for k = 2–9. **C** Consensus clustering heat map for k = 2 in HCC samples. **D** Heat map plot showed hot tumor signature genes were enriched in hot tumor samples. **E** NAPSB was significantly overexpressed in hot tumors. **F** The expression of NAPSB was positively correlated with immune checkpoint molecules expression levels. **G** Representations (left) and quantification of IHC (right) positive areas of PD-L1 in NAPSB-high and NAPSB-low HCC tissues. **H** Correlations between NAPSB and the enrichment scores of several therapeutic signatures. **I** Differences in enrichment scores of IFN-γ-signature, APM-signal, EGFR-ligands and hypoxia between NAPSB subgroups. **J** Differences in enrichment scores of PPARG network, β-catenin signaling pathway, VEGFA and IDH1 between NAPSB subgroups. **K** The proportion of immune response to immunotherapy of NAPSB subgroups in GSE91061. **L** NAPSB was highly expressed in the CR/PR group in GSE91061. CR/PR: Complete and partial response. PD/SD: Progressive and stable disease. ns, no significance; *, *p*-value < 0.05; **, *p*-value < 0.01; ***, *p*-value < 0.001; ****, *p*-value < 0.0001

Unsupervised clustering was conducted to classified HCC samples into hot tumors and cold tumors based on the hot tumor signature genes (Additional file 1: Supplementary Table 14; Fig. 4A–D) [30]. The expression of NAPSB was compared between hot and cold tumors, and we found that it was overexpressed in hot tumors (Fig. 4E), suggesting that NAPSB could play a role in distinct hot/cold tumor states and be associated with therapeutic response to immunotherapy. The same methods were used to validate the above results in the ICGC cohort (Additional file 5: Supplementary Fig. 3A–E).

In addition, NAPSB expression was found to be positively correlated with BTLA, CTLA-4, IDO1, LAG-3, PD-1, PD-L1, TIGIT and TIM-3 expression (Fig. 4F), which were well-known predictors of response to immunotherapy. Blockade of the interaction between PD1 and its ligands PD-L1 is the most important immunotherapy currently [40]. The IHC staining showed consistent correlation between NAPSB and PD-L1 expression in the Zhongnan cohort (Fig. 4G). Also, the enrichment scores of therapeutic signatures that predict clinical response were compared in NAPSB subgroups. As exhibited in Fig. 4H, J, NAPSB was negatively correlated with the enrichment scores of PPARG network, β-catenin signaling pathway, VEGFA and IDH1, all of which were immunosuppressive gene signatures [41–44]. However, in the NAPSB-high group, immunotherapy-positive pathways such as IFN-γ-signature, APM-signal, EGFR-ligands, hypoxia and KDM6B were activated (Fig. 4I) [45–49], indicating high NAPSB is beneficial to immune activation and immunotherapy response. These observations were also validated using ICGC samples (Additional file 5: Supplementary Fig. 3F–H).

The last but important, the role of the NAPSB in predicting the immune checkpoint blockade (ICB) response was explored in two immunotherapy-related melanoma cohorts. In the GSE91061, we found the ICB response rates were obviously higher in the NAPSB-high group than in the NAPSB-low group (Fig. 4K) and the expression of NAPSB was significantly high in the response group (Fig. 4L). Similar results were observed in the GSE78220 cohort (Additional file 5: Supplementary Fig. 3I). These evidences reconfirmed that NAPSB may be a valuable predictor of immunotherapy response across cancers.

### NAPSB was associated with increased sensitivity to chemotherapy

**Fig. 5:**
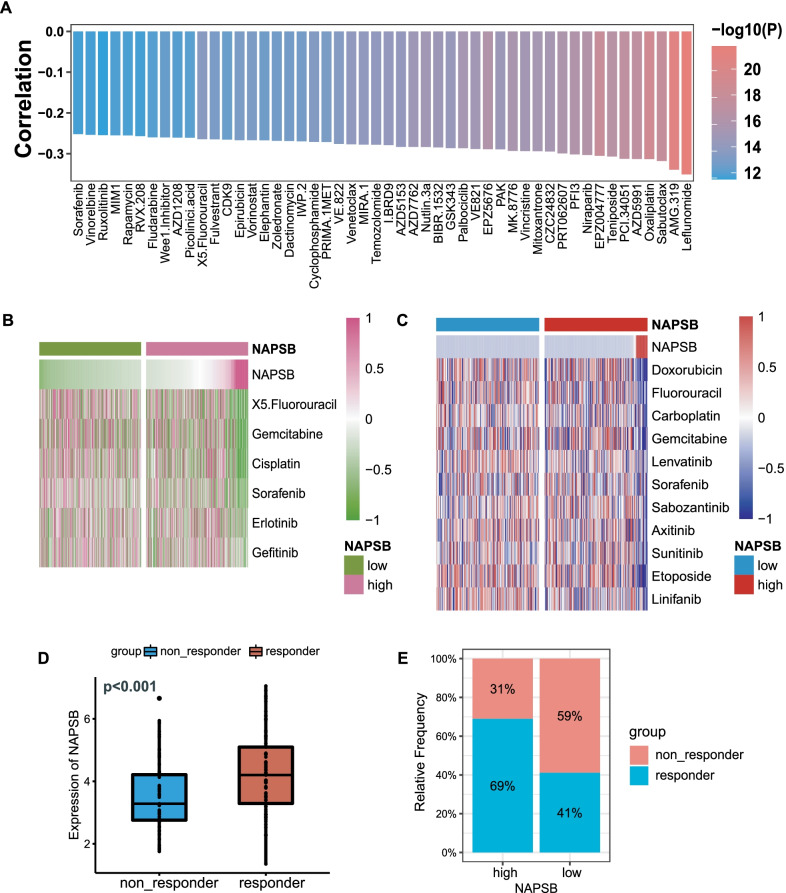
The role of NAPSB in chemotherapy response. **A** Bar plot exhibiting the Spearman’s correlation between NAPSB and the IC50 of drugs (the top 50 drugs in order of p-value from smallest to largest) in GDSC. **B** Correlations between NAPSB and the IC50 of the frequently used drugs for advanced patients with HCC in CTRP, including doxorubicin, fluorouracil, carboplatin, gemcitabine, lenvatinib, sorafenib, sabozantinib, axitinib, sunitinib, etoposide and linifanib. **C** Correlations between NAPSB and the IC50 of the commonly used drugs for advanced patients with HCC in GDSC, including fluorouracil, gemcitabine, cisplatin, sorafenib, erlotinib and grfitinib. **D** NAPSB was highly expressed in CR/PR group of transarterial chemoembolization (TACE) therapy in GSE104580. **E** The proportion of response to TACE of NAPSB subgroups in GSE104580. ns, no significance; *, *p*-value < 0.05; **, *p*-value < 0.01; ***, *p*-value < 0.001; ****, *p*-value < 0.0001

Using data from GDSC and CTRP, the role of NAPSB in chemotherapy sensitivity was analyzed. Intriguingly, NAPSB expression was negatively associated with IC50 of most agents in GDSC and CTRP (Fig. 5A and Additional file 6: Supplementary Fig. 4; Additional file 1: Supplementary Table 15), supporting that NAPSB can enhance the therapeutic response to chemotherapy. Two heat maps (Fig. 5B, C) showed that the IC50 of some commonly used drugs for patients with HCC was lower in the NAPSB-high group in GDSC and CTRP databases, respectively. Results above speculated that high expression of NAPSB is beneficial to the sensitive response of chemotherapy.

Thereafter, by analyzing GSE104580, a HCC cohort of TACE, we found the expression of NAPSB was significantly higher in the TACE response group (Fig. 5D), and the response rates were obviously higher in the NAPSB-high group than in the NAPSB-low group (Fig. 5E). This data further illustrated that high expression of NAPSB may be beneficial to chemotherapy response.

### Association of NAPSB with cell death of tumor cells

**Fig. 6:**
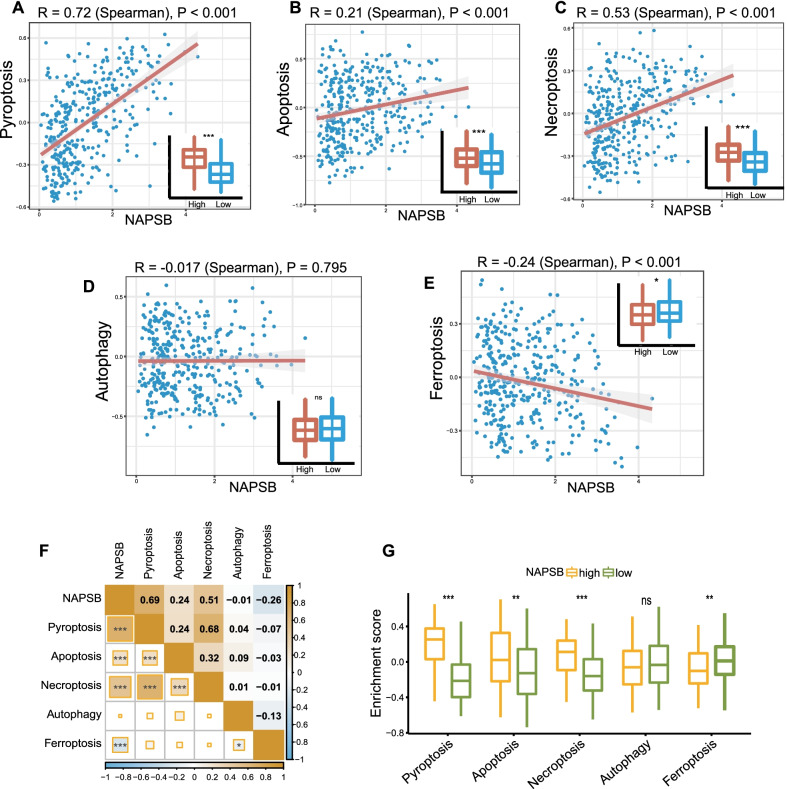
Correlations between NAPSB and the enrichment scores of several cell death signatures in the TCGA **A–E** and ICGC **F** and **G** cohorts. NAPSB expression was positively correlated with pyroptosis (**A**; R = 0.72, *P* < 0.001), apoptosis necroptosis (**B**; R = 0.21, *P* < 0.001) and necroptosis (**C**; R = 0.53, *P* < 0.001). **D** Autophagy had no correlation with NAPSB expression (R = − 0.017, *P* = 0.795). **E** NAPSB was negatively correlated with ferroptosis (R = − 0.24, *P* < 0.001). **F** Correlations between NAPSB and several forms of cell death in the ICGC cohort. **G** The enrichment scores of pyroptosis, necroptosis and apoptosis in the NAPSB-high groups were markedly higher than the NAPSB-low groups. However, ferroptosis score was lower in the NAPSB-high group than in the NAPSB-low group, and autophagy score had no significant difference between NAPSB subgroups. Spearman coefficients were used to explore the correlations. ns, no significance; *, *p*-value < 0.05; **, *p*-value < 0.01; ***, *p*-value < 0.001; ****, *p*-value < 0.0001

Given that cell death had been reported in recent years to play a significant role in tumor therapy [50], we investigated the association between NAPSB and various forms of cell death, including pyroptosis, necroptosis, apoptosis, autophagy and ferroptosis. As showed in Fig. 6A–C, E, NAPSB expression was markedly correlated with pyroptosis, apoptosis and necroptosis, but negatively correlated with ferroptosis. Autophagy had no correlation with NAPSB expression (Fig. 6D). We also validated these findings with the ICGC cohort, and consistent results were obtained (Fig. 6F, G). Among the above results, the correlation between NAPSB and pyroptosis was the most significant. Results above inferred that NAPSB may have a beneficial effect on immunotherapy and chemotherapy responses by promoting PANoptosis in tumor therapy.

## Discussion

In this study, the potential biological functions of NAPSB have been comprehensively explored for the first time in HCC. By analyzing the data from multiple public databases and the Zhongnan cohort, our study obtained consistent results as previous research: NAPSB was downregulated in HCC [15]. Tan et al.’s study showed NAPSB was upregulated in PDAC and related to CD4 + T Cell infiltration [14]. Additionally, upregulation of NAPSB was also found in pre-eclampsia, a status of highly inflammatory activity [51]. It was expected that NAPSB overexpressed under inflammatory conditions. In line with these results, enrichment analyses in this study showed the genes most related to NAPSB were enriched in immune cell receptor signaling pathway and inflammatory response in our study.

A more important part of this study was to comprehensively clarify the immunological role of NAPSB in HCC immune microenvironment. MHC molecules represent antigen presentation and processing capacity, and chemokines and receptors recruit effector TIICs [52, 53], which may upregulate the activities of the cancer-immunity cycle subsequently [54]. In our study, NAPSB was found to be positively correlated with these immunomodulators, suggesting that NAPSB promoted immune activation, which was consistent with the results of enrichment analyses above. In addition, NAPSB expression had a positive correlation with the abundance of immune cells. Currently, the prognosis of HCC is known to be related to the infiltration and activation of immune cells [55, 56], whose presence participates in an inflamed TME [57, 58], supporting the observation that NAPSB can stimulate the immune response in the TME and play an anti-tumor role in HCC, thereby prolonging survival. This could also be used to explain the results of this study: high expression of NAPSB was associated with better prognosis of HCC. Additionally, we observed NAPSB was positively related to the TIS, as well as several critical steps of the cancer-immunity cycle.

. Since both TIS and cancer-immunity cycle reflect the T cell infiltration and anti-cancer immune response of human body [29, 39], these results reaffirmed and extended the close relationship between NAPSB and an immune-hot and inflamed TME.

T-cell infiltration, molecular characteristics of immune activation and anti-tumor response are characteristics of hot tumors [59, 60], so we speculated NAPSB can play a role in distinct hot/cold tumor states based on the above results. Here, NAPSB was highly expressed in hot tumor samples consistently. Not only that, NAPSB was significantly positively correlated with ICB therapeutic targets, such as PD-L1, PD-1 and CTLA-4. Better clinical response to ICB is another character of hot tumors due to more active immune molecules [61]. Together, NAPSB could distinguish between hot and cold tumors, and facilitate immunotherapeutic responses. Meanwhile, we found that samples in the NAPSB-high group were activated in immune-activated pathways, such as IFN-γ signature, which had been revealed to contributing to an inflamed TME and resulting in better clinical responses to immunotherapy. These results not only demonstrated that NAPSB can improve the immunotherapy response, but also reconfirmed the role of NAPSB in activation of immune activity as discussed above.

The main treatments for advanced HCC are still chemotherapy and targeted drugs, among which first-line drugs include doxorubicin, fluorouracil and sorafenib, etc. [2], improving the five-year survival rates of patients with HCC [62]. TACE is a treatment for liver cancer often applying doxorubicin or cisplatin as intra-arterial injection agents [63]. In our study, we proved that NAPSB was negatively correlated with IC50 of a variety of commonly used drugs, but overexpressed in TACE responders as expected, strongly inferring high NAPSB expression can improve the sensitivity of chemotherapy. NAPSB may be utilized as a promising predictive marker for chemotherapy since drug resistance is prevalent at present [7, 64]. Recently, studies have focused on the interactions between tumor cell death and sensitivity or resistance of anticancer therapy. For instance, Makin et al. proposed that apoptosis was the predominant form of regulated cell death, and was responsible for tumor therapies [65]. Carina et al.’s study revealed sorafenib therapy induced pyroptosis in MΦ and thereby enhanced the response of NK-cell against HCC tumors [66]. Instead, autophagy, this cell death form participates in the progression of HCC and the resistance of HCC cells to sorafenib [37, 67]. In our study, we revealed NAPSB was positively correlated with PANoptosis, but had no correlation with autophagy, suggesting that NAPSB may promote PANoptosis to improve the sensitivity of chemotherapy.

Despite these findings, there is existing the limitation that the study was primarily carried out using bioinformatics methods. To remedy this deficiency, the main conclusions of this study were confirmed by several methods and external validation. For instance, differential expression of NAPSB in HCC and normal tissue has been verified in multiple cohorts; the association of NAPSB with immune infiltration was demonstrated by ssGSEA and six other independent algorithms.

## Conclusions

In conclusion, our study is the first comprehensive analysis to demonstrate that NAPSB could shape an immuno-hot and inflamed TME in HCC; NAPSB could be considered a predictor of disease-free and progression-free survival outcomes in patients with HCC; NAPSB can also predict the clinical response to ICB and chemotherapy. These findings will provide important insights for the development of cytokine-based therapy for cancer treatment.

## Supplementary Information

**Additional file 1**.** Supplementary Tables 1-4 and 7-15**.** Table S1**. 50 immunomodulators.** Table S2**. Effector genes of TIICs.** Table S3**. Gene sets of therapeutic signatures.** Table S4**. Gene sets of cell death signatures.** Table S7**. Co-Expressed genes of NAPSB.** Table S8**. Upregulated expressed genes between NAPSB subgroups.** Table S9**. Common genes of co-expressed genes and upregulated DEGs.** Table S10**. Functional annotation and pathway analysis for common genes (Gene Ontology-Biological process).** Table S11**. Functional annotation and pathway analysis for common genes (KEGG pathways).

**Additional file 2**.** Supplementary Tables 5-6**.** Table S5**. Correlation of clinicopathologic characteristics and NAPSB in TCGA-LIHC cohort.** Table S6**. Correlation of clinicopathologic characteristics and NAPSB in ICGC-LIRI-JP cohort.

**Additional file 3**.** Fig. S1**. (A) Univariate Cox regression analyses of NAPSB levels, age, gender and tumor stage for OS, DFI and PFI in the TCGA cohort. (B) Multivariate Cox regression analyses of NAPSB levels with age, gender, and tumor stage for DFI and PFI in the TCGA cohort

**Additional file 4**.** Fig. S2**. High NAPSB was associated with an inflamed TME among patients with HCC from the ICGC cohort. (A) Expression levels of fifty immunomodulators (MHC, immunostimulators, chemokines and receptors) in the NAPSB-high and NAPSB-low groups. (B) Distribution of stromal score, immune score and estimate score calculated using the ESTIMATE algorithm in the NAPSB-high and NAPSB-low groups. (C) Correlation between NAPSB and tumor purity using the ESTIMATE algorithm. (D) Different expression of 28 tumor-associated immune cells calculated with the ssGSEA algorithm between NAPSB subgroups. (E) Correlation between NAPSB and the infiltration levels of six types of TIICs (CD8+ T cells, CD4+ T cells, NK cells, B cells, dendritic cells and macrophages), which were calculated using six independent algorithms. (F) Correlation between NAPSB expression and the effector genes of the above immune cells. (G, H) Correlations between NAPSB and the T cell inflamed score, and the individual genes included in the T cell inflamed signature. ns, no significance; *, p-value < 0.05; **, p-value < 0.01; ***, p-value < 0.001; ****, p-value < 0.0001.

**Additional file 5**.** Fig. S3**. NAPSB was correlated with hot tumor state and improved response to immunotherapy in the ICGC cohort. (A) Consensus clustering cumulative distribution function (CDF) for k = 2-9. (B) Relative change in area under CDF curve for k = 2-9. (C) Consensus clustering heat map for k = 2 in HCC samples. (D) Heat map plot showed hot tumor signature genes were enriched in the hot tumor samples. (E) NAPSB was significantly overexpressed in the hot tumors. (F) The expression of NAPSB was positively correlated with immune checkpoint molecules expression levels. (G) Differences in enrichment scores of IFN-r-signature, APM-signal, EGFR-ligands and hypoxia between NAPSB subgroups. (H) Differences in enrichment scores of PPARG network, β-catenin signaling pathway, VEGFA and IDH1 between NAPSB subgroups. (I) The proportion of immune response to immunotherapy of NAPSB subgroups in GSE78220. CR/PR: Complete and partial response. PD: Progressive disease. ns, no significance; *, p-value < 0.05; **, p-value < 0.01; ***, p-value < 0.001; ****, p-value < 0.0001.

**Additional file 6**.** Fig. S4**. Bar plot exhibiting Spearman correlation between NAPSB and the IC50 of drugs (the top 100 drugs in order of p-value from smallest to largest) in CTRP.

## Data Availability

Publicly available datasets were analyzed in this study. These data can be found here: https://xenabrowser.net/datapages/, https://icgc.org/, and https://www.ncbi.nlm.nih.gov/geo/. The supplementary material for this article can be found online. All processed data and R codes used in this study can be obtained from the corresponding author on reasonable request.
