# Supplementary material for: NAPSB as a predictive marker for prognosis and therapy associated with an immuno-hot tumor microenvironment in hepatocellular carcinoma
Source: BMC Gastroenterol. 2022 Aug 20;22:392. doi: 10.1186/s12876-022-02475-8 (PMC9392949; doi:10.1186/s12876-022-02475-8)
Supplement: Supplementary file 2 — Additional file 2. Supplementary Tables 5-6. Table S5. Correlation of clinicopathologic characteristics and NAPSB in TCGA-LIHC cohort. Table S6. Correlation of clinicopathologic characteristics and NAPSB in ICGC-LIRI-JP cohort. [file 12876_2022_2475_MOESM2_ESM.docx]

**Supplementary Table 5.** Correlation of clinicopathologic characteristics and NAPSB in TCGA-LIHC cohort.

| **Characteristics** | **N** | **Percent (%)** | **NAPSB level**  **Low High** | | ***P***  **Chi squared test** |
| --- | --- | --- | --- | --- | --- |
| **Total cases** | 369 | 100 | 184 | 185 |  |
| **Gender** |  |  |  |  | 0.282 |
| Male | 249 | 67.48 | 129 | 120 |  |
| Female | 120 | 32.52 | 55 | 65 |  |
| **Age** |  |  |  |  | 0.251 |
| ≤60 | 177 | 47.97 | 83 | 94 |  |
| >60 | 191 | 51.76 | 101 | 90 |  |
| **Clinical stage** |  |  |  |  | 0.110 |
| I | 171 | 46.34 | 76 | 95 |  |
| II | 86 | 23.31 | 48 | 38 |  |
| III | 83 | 22.49 | 46 | 37 |  |
| IV | 5 | 1.36 | 4 | 1 |  |
| **Pathology T stage** | |  |  |  | 0.218 |
| T1 | 182 | 49.32 | 83 | 99 |  |
| T2 | 94 | 25.47 | 52 | 42 |  |
| T3 | 78 | 21.14 | 44 | 34 |  |
| T4 | 13 | 3.52 | 5 | 8 |  |
| **Pathology N stage** |  |  |  |  | 0.306 |
| N0 | 250 | 67.75 | 123 | 127 |  |
| N1 | 4 | 1.08 | 3 | 1 |  |
| **Pathology M stage** |  |  |  |  | 0.347 |
| M0 | 265 | 71.82 | 136 | 129 |  |
| M1 | 4 | 1.08 | 3 | 1 |  |
| **Vascular invasion** |  |  |  |  | 0.219 |
| None | 206 | 55.83 | 98 | 108 |  |
| Mico | 93 | 25.20 | 54 | 39 |  |
| Macro | 14 | 3.79 | 8 | 6 |  |
| **Adjacent hepatic tissue inflammation extent** |  |  |  |  | 0.257 |
| None | 117 | 31.71 | 69 | 48 |  |
| Mild | 99 | 26.83 | 53 | 46 |  |
| Severe | 18 | 4.88 | 7 | 11 |  |

**Supplementary Table 6.** Correlation of clinicopathologic characteristics and NAPSB in ICGC-LIRI-JP cohort.

| **Characteristics** | **N** | **Percent (%)** | **NAPSB level**  **Low High** | | ***P***  **Chi squared test** |
| --- | --- | --- | --- | --- | --- |
| **Total cases** | 231 | 100 | 115 | 116 |  |
| **Gender** |  |  |  |  | 0.913 |
| Male | 170 | 73.59 | 85 | 85 |  |
| Female | 61 | 26.41 | 30 | 31 |  |
| **Age** |  |  |  |  | 0.605 |
| ≤60 | 49 | 21.21 | 26 | 23 |  |
| >60 | 182 | 78.79 | 89 | 93 |  |
| **Clinical stage** |  |  |  |  | 0.738 |
| I | 36 | 15.58 | 15 | 21 |  |
| II | 105 | 45.45 | 54 | 51 |  |
| III | 71 | 30.76 | 37 | 34 |  |
| IV | 19 | 8.23 | 9 | 10 |  |
| **History of malignancy** |  |  |  |  | 0.230 |
| No | 201 | 87.01 | 97 | 104 |  |
| Yes | 30 | 12.99 | 18 | 12 |  |
| **Tumor history of first-degree relatives** |  |  |  |  | 0.003 |
| No | 143 | 61.90 | 60 | 83 |  |
| Yes | 73 | 31.60 | 43 | 30 |  |
